# Spatial clustering and its effect on perceived clustering, numerosity, and dispersion

**DOI:** 10.3758/s13414-016-1100-0

**Published:** 2016-05-03

**Authors:** Marco Bertamini, Michele Zito, Nicholas E. Scott-Samuel, Johan Hulleman

**Affiliations:** Department of Psychological Sciences, University of Liverpool, Eleanor Rathbone Building, Liverpool, L69 7ZA UK; Department of Computer Science, University of Liverpool, Liverpool, UK; School of Experimental Psychology, University of Bristol, Bristol, UK; Department of Psychological Sciences, University of Manchester, Manchester, UK

**Keywords:** Numerosity, Occupancy, Graph theory, Clustering

## Abstract

Human observers are able to estimate the numerosity of large sets of visual elements. The occupancy model of perceived numerosity in intermediate numerical ranges is based on overlapping regions of influence. The key idea is that items within a certain range count for less than their actual numerical value and more so the closer they are to their neighbours. Therefore occupancy is sensitive to the grouping of elements, but there are other spatial properties of  configurations that could also influence perceived numerosity, such as: area of convex hull, occupancy area, total degree of connectivity, and local clustering For all indices apart from convex hull, we varied the radius of the area that defined neighbours. We tested perceived numerosity using a fixed number of elements placed at random within a circular region. Observers compared two patterns (presented in two intervals) and chose the one that appeared more numerous. The same observers performed two other separate tasks in which they judged which pattern appeared more dispersed or more clustered. In each pair of images, the number was always the same (22, 28, 34, or 40 items), because we were interested in which "appeared" more numerous on the basis of spatial configuration. The results suggest that estimates of numerosity, dispersion, and clustering are based on different spatial information, that there are alternative approaches to quantifying clustering, and that in all cases clustering is linked to a decrease in perceived numerosity. The alternative measures have different properties and different practical and computational advantages.

Human observers have the ability to judge numerosity of large sets of elements with ease, something that has led to introduction of the term "number sense" (Dehaene, 1992). Being able to estimate the numerosity of a cluster of items has clear behavioural advantages, but for this estimation process to be maximally useful, it should be fast and it should apply to large sets where counting the items is impossible or impractical.

That observers can estimate numerosity of large sets with brief presentation is not disputed (Izard & Dahaene, 2008). Many researchers have focused on specific aspects of the underlying mechanism. One important finding is that numerosity judgments can be biased by a number of irrelevant dimensions, such as the size of the elements (Ginsburg & Nicholls, 1988; Hurewitz, Gelman, & Schnitzer, 2006; Sophian, 2007; Tokita & Ishiguchi, 2010), the contrast and contrast polarity (Tibber, Greenwood, & Dakin, 2012), the regularity of the configuration (Frith & Frith, 1972; Ginsburg, 1976, 1991), and the total area (Hurewitz, Gelman, & Schnitzer, 2006; Tokita & Ishiguchi, 2010). An issue for which there is debate is whether perception of numerosity and perception of density are served by the same mechanism (Dakin, Tibber, Greenwood, & Morgan, 2011; Tibber, Greenwood, & Dakin, 2012; Burr & Ross, 2008).

We focus on what makes sets of elements appear more or less numerous when the elements themselves are identical and the set size is fixed. The only variables of interest were the spatial properties of the distribution of the elements. It is well known that the configuration of elements can bias judgments of numerosity. For instance, Ginsburg (1976) coined the term *Jacob*'*s illusion*. In the Bible, Jacob instructed his servants to send some sheep as a gift to his brother; to make the flock appear more numerous, he instructed them to drive the sheep forward in groups. Frith and Frith (1972) used the term *Solitaire illusion* to refer to a related phenomenon: elements appear to be more numerous when they form a coherent group on the basis of Gestalt grouping. The best-known effect of configuration, however, is probably that of regularity (Cousins & Ginsburg, 1983; Ginsburg 1976, 1980). To observe the regularity-random numerosity illusion, one needs to compare perceived numerosity for elements that are regularly spaced and elements that are positioned at random: the regularly spaced elements appear more numerous. In summary, many studies have shown that the type of configuration of elements influences perceived numerosity. It is necessary to study in detail these configurational effects, and to do so there is a need to define the relevant Gestalt structures (Wagemans et al., 2012). What is referred to as regularity in the regularity-random numerosity illusion may be better described as an effect of spacing, because in the more regular arrays the elements do not come close to each other. It also has been observed that elements occupying a wider region appear more numerous than those confined to a smaller region (Bevan et al. 1963; Binet, 1890; Ponzo, 1928) and that grouping affects the time taken to count a configuration. Observers count small groups of dots in turn and then sum these to arrive at the total (Van Oeffelen & Vos 1982; 1984).

However, it is not easy to integrate all the evidence in a simple model. One possibility is that clustering of elements plays a critical role (Ginsburg & Goldstein, 1987); this idea has led to the proposal of the occupancy model, which we now discuss in more detail.

## Measuring clustering

The idea that elements interact with neighbouring elements and that this clustering affects perceived numerosity has been developed by Vos, Van Oeffelen, Tibosch, & Allik (1988). This led to the development of the occupancy model (Allik & Tuulmets, 1991). Although each element may have a physical extent, elements are hypothesised to have regions of influence around them. The union of all these regions of influence defines an area. According to the occupancy model, the size of this area is used as the basis for judging numerosity, and configurations with larger occupancy value are chosen as more numerous.

The definition of the region of influence is of course important. One can imagine many ways to model it, and the original proposal suggested the use of a monotonically decreasing function around the element. This has been included in formal models of grouping by proximity (van Oeffelen & Vos, 1982; Compton & Logan, 1993). For simplicity, in this work we adopt a thresholding mechanism: the region of influence is a circle with a fixed radius. Intersecting regions decrease the numerosity estimate, while nonintersecting ones (no matter how far apart they are) do not have such effect on the overall numerosity estimate.

The role of regularity and clustering has been studied in several papers (Allik & Tuulmets, 1991; Burgess & Barlow, 1983; Ginsburg & Goldstein, 1987). A related phenomenon is the effect of connecting lines between elements. Franconeri, Bemis, and Alvarez (2009) found that participants underestimated the number of objects that were grouped by lines, relative to disconnected objects. In another recent study, Valsecchi, Toscani, and Gegenfurtner (2013) have shown how the point of subjective equality depends both on clustering and on eccentricity. Their methodology was based on an adaptive procedure and on the fitting of psychometric functions from which an estimate of the point of subjective equality can be obtained. The implementation of clustering was based on constraining the centre-to-centre distance of the dots. For a subset of dots, the distance from the nearest dot had a range that could be short, medium, or large. Valsecchi, Toscani, and Gegenfurtner (2013) confirmed that greater clustering reduces perceived numerosity and suggested crowding (Bouma, 1970; Pelli & Tillman, 2008) as a possible explanation. More recently Anobile, Turi, Cicchini, and Burr (2015) also suggested a role for crowding in numerosity estimation, based on the effect of eccentricity.

The occupancy model uses total area as a way of measuring the interaction between elements—in particular, the effect of proximity. However, proximity and clustering of a configuration of elements can be measured in a number of ways. If the local interactions are due to the formation of binary links (in the sense that they have values of true or false only) between elements, perhaps an alternative model should try to count these links more directly. Borrowing the terminology of graph theory, these are edges between nodes and the total degree of a configuration is the total number of edges. On the other hand, local clustering may be the more important factor: local groups form wholes, and these wholes affect perceived numerosity because they will be relatively low in number (compared to the individual elements). If local clusters are important, we can compute a local clustering index and then average that for the whole configuration.

In this study, we focus on four indices: convex hull, occupancy area, total degree, and local clustering. They will be defined and discussed in more detail in the next section.

## Measuring structure within random configurations

**Fig. 1:**
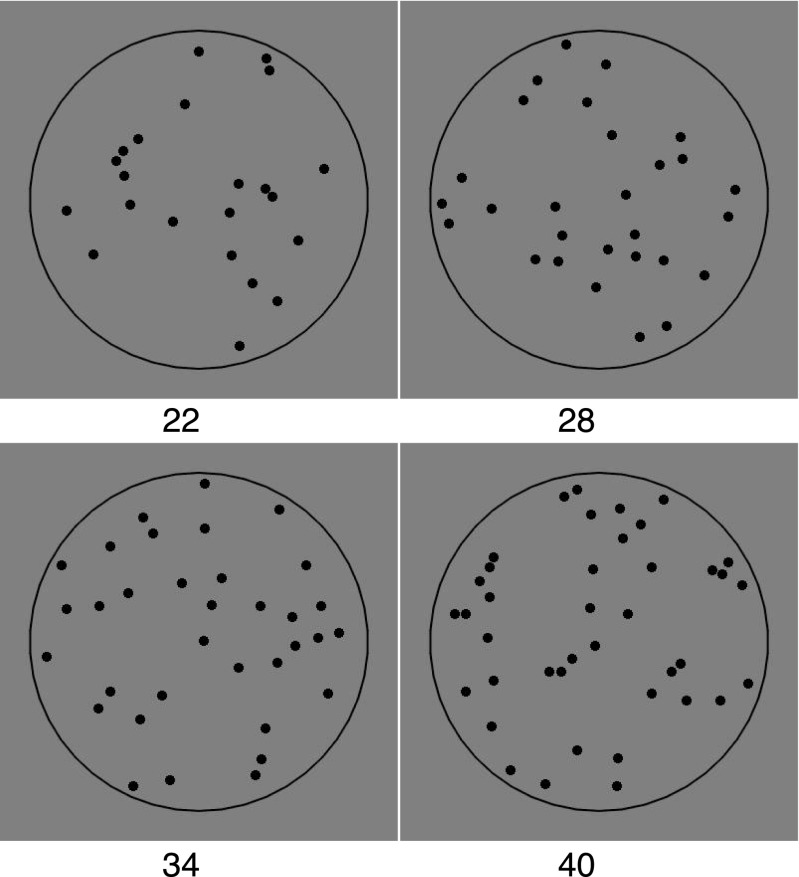
Examples of stimuli with 22, 28, 34, and 40 elements within a circular contour. The only constraint on position was that the elements could not overlap

We are interested in what guides observers in their judgments of spatial properties of a large set of elements. Therefore, we used sets composed of identical circular elements. In what follows, the size of an element refers to its radius. The numerosity was chosen to be outside the subitizing range (i.e., >4) and also to be too large for observers to count each element in turn within the presentation time. Specifically, our configurations had between 22 and 40 elements (Fig. 1). In a two-interval forced-choice task, participants selected which interval appeared to have more elements. The two intervals always had the same number of elements, so there was no objectively correct or incorrect answer. Instead, we were interested in differences in perceived numerosity. We opted for a two-interval procedure, because we wanted both configurations to be scanned foveally and for an equal amount of time (Kingdom & Prins, 2010).

In separate blocks of trials, the same observers performed two other tasks. Observers were asked which of the two configurations of stimuli appeared less dense and more dispersed (dispersion task) and which appeared more clustered (clustering task). To explain dispersion, the experimenter used both the terms dispersion and density (observers were told that a dispersed configuration is the opposite of a dense one). To explain clustering, the experimenter also used the term local groupings. The order of the blocks was counterbalanced.

As noted in the discussion of the literature, clustering has been studied by constraining dot location. By contrast, instead of a manipulation of the properties of the stimuli, the strategy adopted in this study was to place elements randomly within a circular region of fixed size and then compute different properties of each configuration on a trial-by-trial basis. The only constraint on the placement of the elements was that they could not overlap. Different strategies have different strengths. Introducing constraints may test specific hypotheses, but when comparing the usefulness of different measures, it is essential to start from configurations that are as unconstrained as possible so as not to bias the outcome.

## Description of the different indices

In this section, we describe the four indices that will be used in our analysis. In what follows *S* is the given set of $n\;\left(=\left|S\right|\right)$ points, all within a circle *C* of radius $R$ around the origin. With small $r$ we refer to a critical distance that defines neighbourhoods for each point. ${G}_r$ denotes the geometric network obtained from the given points, connecting points at distance less than $2r$. The point set is denoted by $V\left({G}_r\right)$; the set of its lines is denoted by $E\left({G}_r\right)$.

### Convex hull

A convex set *S* (on a 2-dimensional surface) is a collection of points such that, given any two points *X* and *Y* in *S* , the line segment $XY$ joining the two points lies entirely in *S*. The convex hull of a set of points *S* is the smallest convex set containing all points in *S*. Thus, the convex hull of three noncollinear points on the plane is a triangle, but the convex hull of four noncollinear points also could be a triangle if one of the points is inside the triangle formed by the other three. In general, the convex hull may be visualized as the shape enclosed by a rubber band stretched around the points in *S* (for more examples see en.wikipedia.org/wiki/Convex_hull).

**Fig. 2:**
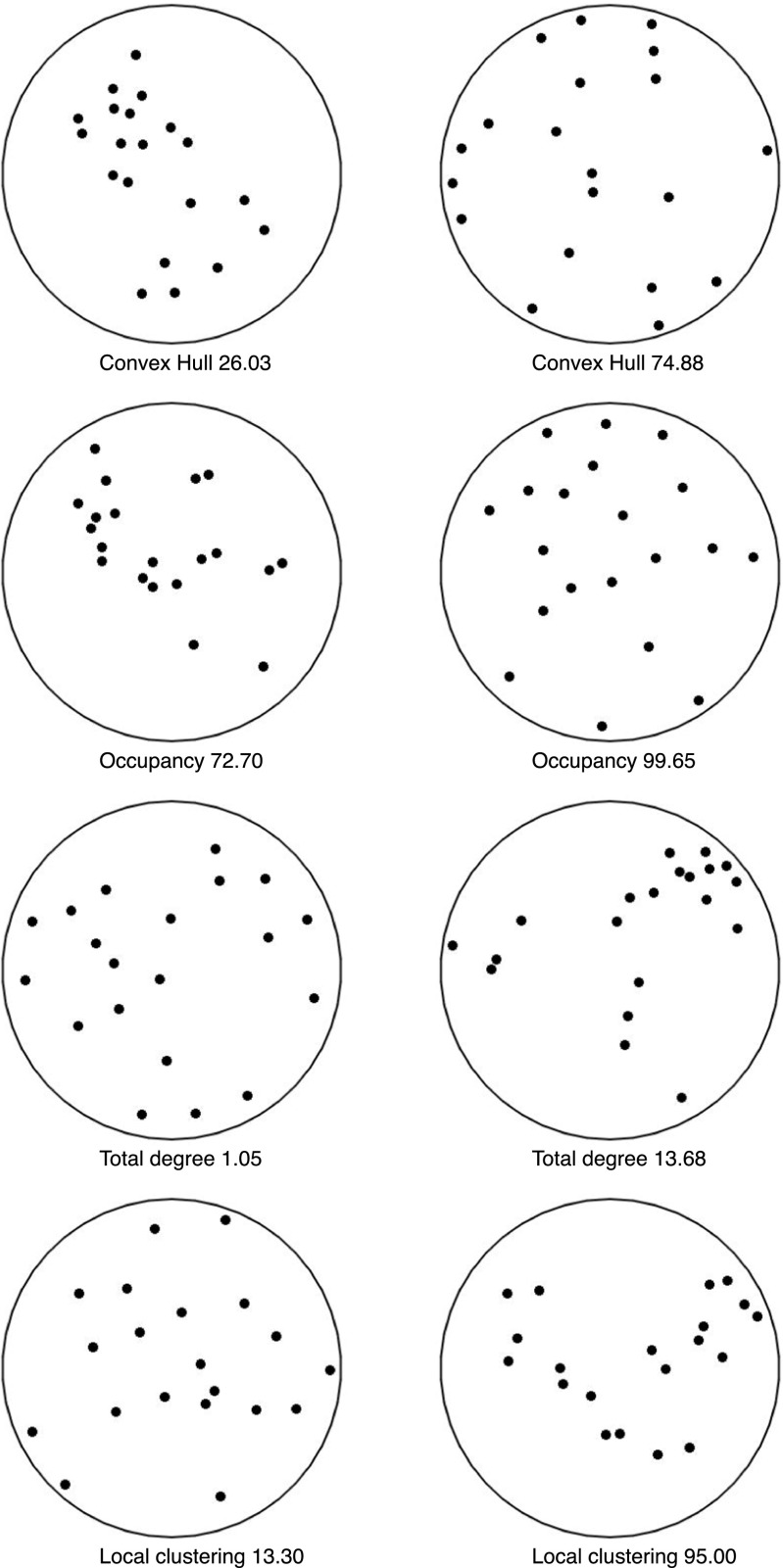
Examples of extreme values for each of the four indices. To illustrate, 20 dots are shown in each example and the parameter r is always 4 times the radius of the dot. The configurations are those with minimum (left) and maximum (right) values from within 10,000 random configurations. For ease of comparison, values are expressed as percentages. For example in the case of the convex hull, the area was divided by the area of the circle. For occupancy, the area was divided by the maximum value (the sum of areas without any overlap). Note a similarity in the comparison between the left and right columns (with a reversed pattern in the top two and in the bottom two)

The area of the convex hull could be used to compare the numerosity of different patterns. Such quantity is upper bounded by *A*=π*R*^*2*^ the area of the circular region in which all points in *S* are located. Intuitively the area depends on how much the points are spread out. Figure 2 (top row) provides an example of two configurations with large and small areas. The value underneath is the area of the convex hull divided by the area of the circle (and expressed as a percentage). Given that perceived numerosity can increase with the size of the display and that the two can be assimilated (Bevan & Turner, 1964), we expect a link between this index and perceived numerosity.

### Occupancy area

As discussed in the introduction, a classic approach for estimating numerosity is based on drawing circles of radius $r$ around the given points and measuring the area of the part of *C* covered by at least one circle. The resulting index is more precise than the convex hull in the sense that configurations sharing the same convex hull may have different occupancy depending on the position of the points. The value of the occupancy depends on the number and relative position of the points as well as on $r$, the radius of influence.

In our study, nine values of the radius were used: 2, 3, 4, 5, 6, 7, 8, 9, and 10 times the size of the element. To compare the index for set sizes of 22, 28, 34, and 40 elements, we divided occupancy area by the maximum occupancy area (i.e., the sum of all regions of influence without any overlap), thus obtaining a proportion. The maximum value of 1.0 indicates that none of the regions of influence overlap, and a value of 0.8 indicates a 20 % overlap for the regions. An example with two extreme cases in provided in Fig. 2 (second row). We expected perceived numerosity to increase with an increase in occupancy area, and the opposite should be true for perceived clustering.

### Total degree

A further option for measuring the distribution of the elements is to use the network's total degree. In general, the total degree is the total number of lines out of any node in the graph. We denote such quantity by *D*, and in symbols we have

$$ D={\displaystyle {\sum}_{u\kern0.5em \in V\left({G}_r\right)} \deg \kern0.5em u} $$

where $\deg u$ counts the adjacencies of vertex *u*.

Note that *D* is proportional to a number of other measures. For instance, the average number of lines out of a node in *G*_*r*_ is just *D*/*n*, whereas the total number of lines in the network is *D*/*2*, because each line contributes twice to *D*. The total degree of a network can be zero, if the nodes are not linked. If the network does not have lines starting and ending at the same node or parallel lines, then the maximum value of $D$ is $n\left(n-1\right)$, when every node of ${G}_r$ is connected to every other. Thus, configurations in which the nodes form groups will have higher values of $D$. Note, however, that $D$ is only partially affected by the number of such groups or the distance between the nodes.

Nine sizes of the radius were used: 2, 3, 4, 5, 6, 7, 8, 9, and 10 times the size of the element. To compare the index for set sizes of 22, 28, 34, and 40 elements, we divided degree by the maximum degree $n\left(n-1\right)$, thus obtaining a proportion. For instance, a value of 0.8 indicated that 80 % of all possible connections were active. We expected perceived numerosity to decrease with an increase in total degree of connectivity.

### Local clustering

The index described in this paragraph provides on possible way of measuring the clustering properties of a set of points. Let ${e}_r(S)$ be the number of lines in the network induced by the set of points *S* using radius $r$. In what follows, we also will omit any reference to the proximity radius when its value is clear from the context. The quantity ${\mathrm{lc}}_v$ defined as

$$ \frac{2\cdot e\left(N(v)\right)}{ \deg \kern0.5em v\cdot \left( \deg \kern0.5em v-1\right)} $$

is called the local clustering coefficient of node *v* (here $N(v)$ is the set of nodes directly connected to node *v*). Index ${\mathrm{lc}}_v$ measures the ratio between the number of lines in the immediate neighbourhood of node *v*, and the maximum possible value for such quantity, which is equal to $\deg v\bullet \left( \deg v-1\right)/2$. Thus, for instance, a node *v* connected to four other nodes all of which also are connected to each other will have maximum local clustering value (one). However, if only three of those nodes are connected to each other, while the fourth one is only connected to *v*, the local clustering of *v* would be $1/2$ as there are only three connections among the neighbours of *v* but the maximum possible value is $\deg v\bullet \left( \deg v-1\right)/2=4\left(4-1\right)/2=6$. One can then define the local clustering of the whole network as the average of these values over the total number of points.

The local clustering uses information about the lines in the network (similar to the total degree) but also about their (local) arrangement. It is easy to check from the definition that the local clustering value ranges between 0 and 1. In particular, paths and cycles have low clustering values, whereas networks formed by clutches of nodes tend to have clustering close to one, irrespective of the number of such clutches.

In the literature, there are various formal methods proposed for measuring grouping and clustering. Starting from the Gestalt idea of grouping by proximity, there has been work on how proximity affects large configurations of dots (Kubovy & Wagemans, 1995) and how it can predict local groups (van Oeffelen & Vos, 1982; Compton & Logan 1993; 1999). A recent paper by Im, Zhong & Halberda (Im et al. 2015 introduced a model relying on a modified k-means clustering algorithm. They concluded that the grouping window has a size of approximately 4 deg of visual angle and confirmed that grouping is inversely proportional to perceived numerosity.

## Methods

### Participants

Twenty-four observers from the University of Liverpool community took part in the study (2 males). The mean age was 19 (range 18-21) years. All had normal or corrected to normal vision. Students received course credits for their time. The order of the tasks (numerosity, dispersion, clustering) was balanced based on a Latin square, creating three groups (N = 8 in each): NDC, DCN, CND.

### Stimuli and procedure

The task was a temporal two-interval forced-choice (2IFC). The number of elements was always the same in the two intervals, and the values were: 22, 28, 34, and 40. There were 50 trials for each value, thus giving a total of 200 trials for each of the tasks (numerosity, dispersion, clustering). The task was preceded by a short practice consisting of four trials. The stimuli were generated so that each observer saw a novel pattern on each trial without repetition.

Elements were randomly placed within a visible circular outline, with a diameter of 240 pixels (6.45 deg of visual angle). The only constraint was that dots could not overlap. In the literature on perception of density often the elements have high-spatial-frequency and are luminance-balanced (Durgin & Hammer, 2001). In this experiment, we used simple black circles with a diameter of 10 pixels (0.27 deg of visual angle). However, there were no differences in luminance between configurations, because in each trial the number of elements was the same in both intervals.

The presentation of the stimuli and the recording of the responses were controlled by a program written in Python using the PsychoPy library (Peirce, 2007). The stimuli were presented on a CRT monitor (resolution 1024 × 768; 75 Hz).

Observers were seated at approximately 80 cm from the monitor in a dark and quiet room. The time between trials was random between 1 and 1.5 sec. The two intervals on each trial were each presented for 520 msec, with a 26-msec black interstimulus interval. After presentation of this pair of stimuli (configurations), observers pressed one of two keys on the keyboard to indicate which appeared to have more elements (numerosity task), which appeared more dispersed (dispersion task), or which appeared to have more clustering (clustering task).

## Results

For each pair of configurations, we computed the difference between the indices by subtracting the value for the first interval from the value of the second interval. This continuous value can be positive or negative depending on which interval had a higher value, and the size of the difference also is informative. Over a large set of trials, the average difference should be zero. Before considering the relationship between the indices and the judgments of numerosity, dispersion, and clustering, we consider some aspects of the stimuli. In the next three paragraphs, we will briefly discuss the effect of radius on the value of the indices, then the relationship between indices, and finally the relationship between the judgments and the indices.

### Importance of radius

**Fig. 3:**
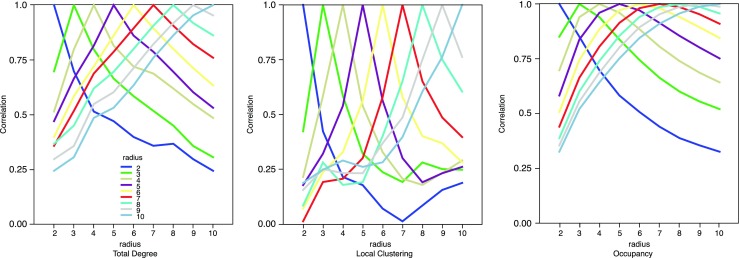
Correlations between values computed with different radii. The peak in the lines is the point where correlation is 1. For all indices, the correlation drops quickly on either side, showing that radius is indeed an important parameter

As mentioned above, apart from the convex hull, all other indices depend on a parameter, which is the region of influence surrounding each element. We varied this as a multiple of the size of the element itself. Therefore, for a radius of 2 the region had twice the radius of the element and an area 4 times as large. Figure 3 shows how each index correlates with itself at different values of this parameter.

### Comparing indices

**Fig. 4:**
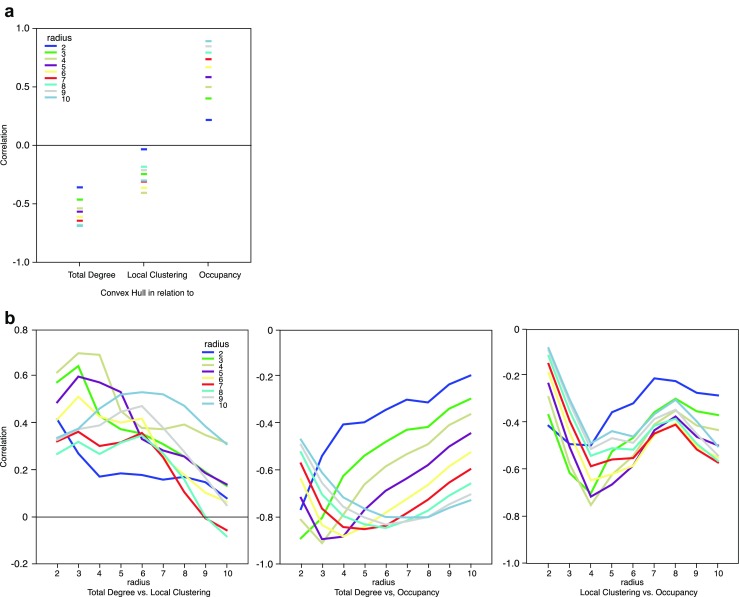
Correlations between values computed with different indices and different radii. (**A**) Convex hull in relation to the other three indices. The relationship is positive with occupancy and negative for the other two. (**B**) Relationship between each pair of indices as a function of radius. Note how correlations range from some high values (close to 1) to values close to zero in other cases. Total degree and local clustering tend to be positively correlated, and both are negatively correlated to occupancy. This is because as area increases clustering decreases

We expected the four indices in this study to be correlated. We computed the correlations for the stimuli that were used in the experiment, and they are presented in Fig. 4. The Convex Hull index does appear to be strongly correlated with Total Degree and with Occupancy, but only when the radius is large. This is logical, especially for occupancy, because with a large radius area increases as the elements are more spaced over the whole region. The lowest correlation is between Convex Hull and Local Clustering. This is likely to be due to the fact that local connectivity can exist within a single close network (small Convex Hull) or within distant subsets (large Convex Hull).

Total Degree, Local Clustering, and Occupancy area measure different aspects of the same physical relationship of clustering of the elements. Therefore, we expected them to correlate. Results on the random patterns used in our experiments confirm this. In particular, Total Degree and Local Clustering are positively correlated: increases in the number of lines in the network push the clustering up as, intuitively, an increased number of lines must populate the neighbourhoods of some nodes. These two indices (Total Degree and Local Clustering), however, are negatively correlated with the Occupancy area as the area is largest when the points are far from each other, *i.e*., when the network has very few lines. Note in particular the complex patterns in some of the panels, suggesting that the indices are qualitatively different in what they measure, in so far that they respond in nonlinear ways to the increasing radius size. In other words, even if correlated they cannot be easily reduced to a single measure.

**Fig. 5:**
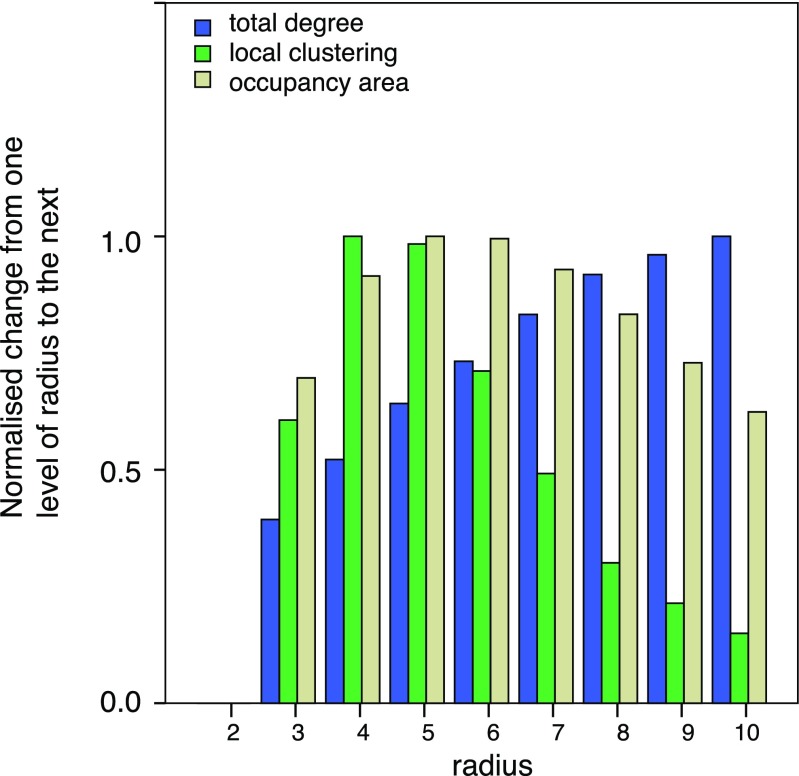
Unsigned, normalised average index changes as a function of the radius. The plots peak differently for each of the three indices (early for local clustering, in the middle for occupancy, and later for total degree)

Figure 5 shows one final property of the indices. We computed the average change in value of the three indices as a function of radius. These changes are plotted unsigned and standardised (to the highest value) for ease of comparison. We note that the plot peaks at a particular value for occupancy and local clustering, whereas it is monotonically increasing for total degree. In the next paragraph, we hypothesise that such peaks might be important in relation to how observers respond to configural information.

### Comparing responses to numerosity, dispersion, and clustering

It is possible that when faced with a large set of elements and a set of questions, observers adopt a strategy of relying on the same visual analysis. The first test therefore was about the correlation between the three responses. We computed the φ coefficient as a measure of association for each observer. The mean values were close to zero as shown in Table 1. It seems that observers used different information to perform the three tasks. There was one exception: the average correlation was greater than zero for the numerosity task and dispersion task (t(23) = 3.86, *p* < 0.01). This correlation was, however, absent when analysed for the separate groups (only the individuals who responded to numerosity first compared to only individuals who responded to dispersion first). Even in the within-subject analysis, despite the fact that the value was greater than zero, the actual correlation value was negative for five individuals (>20 %). Overall, it appears that the link is weak.

**Table 1:** Strength of association between responses to the tasks for each individual (averaged for the whole sample) expressed as φ coefficient

	Average φ value	
Pair of tasks	Within-subjects	Between-subjects
Numerosity and Dispersion	0.071	−0.015
Numerosity and Clustering	0.005	0.082
Dispersion and Clustering	0.050	0.044

In the first computation, the correlation is for responses within each observer. In the second, the correlation is between different observers, relying on the first task (a between design) to exclude any effect of one task on another.

In the following sections, we analyse the relationship between the judgements provided by the observers and the predictions based on the four indices studied in this paper. Because we used a task in which observers had to choose between two intervals, the predictors in our models will be the difference in the values of the index between the two configurations in the two intervals, for each index.

For each of the 200 item pairs, we computed the proportion of participants who chose the second item of the pair for the numerosity, dispersion, and clustering task. Subsequently, we performed three multiple regressions, with the proportions for numerosity, dispersion, and clustering as the dependent variable, respectively. As independent variables, we used the differences on convex hull, occupancy area, local clustering, and total degree of connectivity for the entire range of radii. So, there were a total of 28 potential predictors. Interaction terms were not used. Because this is an exploratory study, we used statistical regression, where independent variables are entered and removed on the basis of their contribution to R^2^. For a predictor to be included in the equation, the improvement of the model had to have a *p* value of 0.05 or better. The criterion for removal from the equation was a *p* value of 0.10 or worse. This procedure takes care of the correlations between the various independent variables, because only the unique contribution to R^2^ made by a particular variable determines whether it will be included or not. Due to the nature of statistical regression, the first independent variable that enters the equation will be the one that most highly correlates with the dependent variable. The regression equation was checked for multicollinearity. When there was multicollinearity between the independent variables (tolerance < 0.20, Menard, 1995), the variable that was entered last was removed from consideration and the statistical regression was repeated.

### Numerosity task

The final equation contained three independent variables and a constant. This model had an adjusted R^2^ of 0.395; *F*(3, 196) = 44.3, *p* < 0.001 (Table 2a).

**Table 2:** Final models for each of the three tasks. IV: independent variable, r: correlation with the response on that task, ΔR^2^: increase in R^2^ when IV is added to regression model

IV	Standardized coefficient	t	Sig.	r	ΔR^2^	Tolerance
A. Final model for numerosity						
Intercept		64.0	<0.001			
Δ Occupancy radius = 5	−0.608	8.9	<0.001	−0.567	0.322	0.658
Δ Total degree radius = 10	−0.391	5.2	<0.001	0.034	0.059	0.534
Δ Convex hull	−0.228	2.7	<0.01	−0.316	0.023	0.440
B. Final model for dispersion						
Intercept		63.5	<0.001			
Δ Total degree radius = 10	−0.295	8.1	<0.001	−0.519	0.269	0.714
Δ Occupancy radius = 5	−0.527	6.1	<0.001	0.017	0.057	0.405
Δ Total degree radius = 5	−0.349	4.4	<0.001	−0.283	0.061	0.361
C. Final model for clustering						
Intercept		60.2	<0.001			
Δ Occupancy radius = 10	0.399	5.0	<0.001	0.649	0.421	0.429
Δ Total degree radius = 10	−0.284	3.7	<0.001	−0.594	0.032	0.462
Δ Local clustering radius = 3	−0.147	2.7	<0.01	−0.303	0.019	0.898

### Dispersion task

The final equation contained three independent variables and a constant. The model had an adjusted R^2^ of 0.378; F(3, 196) = 41.3, *p* < 0.001 (Table 2b).

### Clustering task

The final equation contained three independent variables and a constant. The model had an adjusted R^2^ of 0.464; F(3, 196) = 58.4, *p* < 0.001 (Table 2c).

For all three tasks, the final regression equation consists of three IVs and an intercept. Interestingly, Δ total degree with radius = 10 makes an appearance in all three tasks. For the dispersion task, it is the single IV with the highest correlation and for the clustering task it shows a very high correlation as well. For the numerosity task, however, Δ Total Degree radius = 10 is only entered into the equation, because it improves the predictive value of Δ Occupancy radius = 5. For the dispersion task, these roles are reversed. It also can be seen that there are unique aspects to each of the three tasks in the sense that the clustering task features Δ Local Clustering index with radius = 3 and the numerosity task features Δ Convex Hull, whereas neither is involved in the dispersion task.

From this analysis, it seems that the different ways of measuring the structure within a stimulus (occupancy, total degree, local clustering, convex hull) capture different task relevant aspects. Moreover, the quality of the estimates based on occupancy, local clustering, or total degree depends on the chosen influence radius. Small radii lead to influence regions that are too small to be useful. Large radii lead to the definition of influence regions that have very large overlap and therefore may lose important information about the relative element positions. This leads to a natural question: which radius should be used? While our study is unable to provide a definite answer to this issue, we conjecture that the optimal choice corresponds to a point providing in some sense the best possible estimate with the minimum amount of noise. Figure 5 supports this claim. The data plotted in Fig. 5 can be interpreted as measuring the average rate of change in each the three indices as a function of the influence radius. It seems that the best radius for the numerosity task coincides with the point of the peak in the plot for the occupancy index in Fig. 5. This peak is the point at which the average rate of change starts decreasing.

### Analysis of the first block

As an additional check, we reran the regressions on a subset of the data. Because of the balanced repeated measures design, this second analysis refers only to responses to the first task and therefore is based on one-third of the available data. This has the advantage of removing the possibility that observers adjusted to one question on the basis of the other question, although there is a considerable loss of power.

For the numerosity task the final equation contained two independent variables and a constant. This model had an adjusted R^2^ of .299; *F*(2, 197) = 43.4, *p* < 0.001 (Table 3a). As before, the best predictor was occupancy, although the radius is now six instead of five, and the next factor is total degree. For the density task, the final equation contained three independent variables and a constant. This model had an adjusted R^2^ of 0.394; F(3, 196) = 42.4, *p* < 0.001 (Table 3b). As before, occupancy and total degree swap roles, although for different values of radius. For the Clustering task, the final equation contained one independent variable and a constant. This model had an adjusted R^2^ of 0.142; F(1, 198) = 32.8, *p* < 0.001 (Table 3c). Only one of the three predictors survived. It is likely that power influenced this set of results, but overall they do support a different contribution of different indices for the different tasks.

**Table 3:** Final model for each of the three tasks

IV	Standardized coefficient	t	Sig.	r	ΔR^2^	Tolerance
A. Final model for numerosity						
Intercept		43.9	<0.001			
Δ Occupancy radius = 6	−0.751	6.8	<0.001	−0.536	0.287	0.291
Δ Total degree radius = 5	−0.256	2.3	<0.025	0.377	0.019	0.291
B. Final model for dispersion						
Intercept		122.3	<0.001			
Δ Total degree radius = 8	−0.529	6.7	<0.001	−0.536	0.321	0.493
Δ Convex hull	0.305	3.8	<0.001	0.492	0.022	0.491
Δ Occupancy radius = 5	−0.290	4.0	<0.001	0.193	0.051	0.598
C. Final model for clustering						
Intercept		33.3	<0.001			
Δ Total degree radius = 10	−0.377	5.7	<0.001	−0.377	0.142	1.000

IV, independent variable; r, correlation with the response on that task; ΔR^2^, increase in R^2^ when IV is added to regression model

### Regressions at the individual level

The regressions reported above predict proportion of participants that preferred the second pattern over the first as a function of the difference scores on the Convex Hull, Occupancy, Total Degree, and Local Clustering. However, to allow inferences about these indices at the population level, an analysis is needed that treats participants as a random effect. We therefore fitted logistic regression models to the scores of each individual on each of the three tasks (Clustering, Numerosity, and Dispersion). To select our predictors, we relied on the optimal level as implied by Fig. 5. Specifically we had four predictors: Convex Hull, and the maxima for Occupancy, Total Degree, and Local Clustering from Fig. 5 (Occupancy radius = 5, Total Degree radius = 10, and Local Clustering radius = 4, respectively). All four predictors were entered simultaneously.

**Fig. 6:**
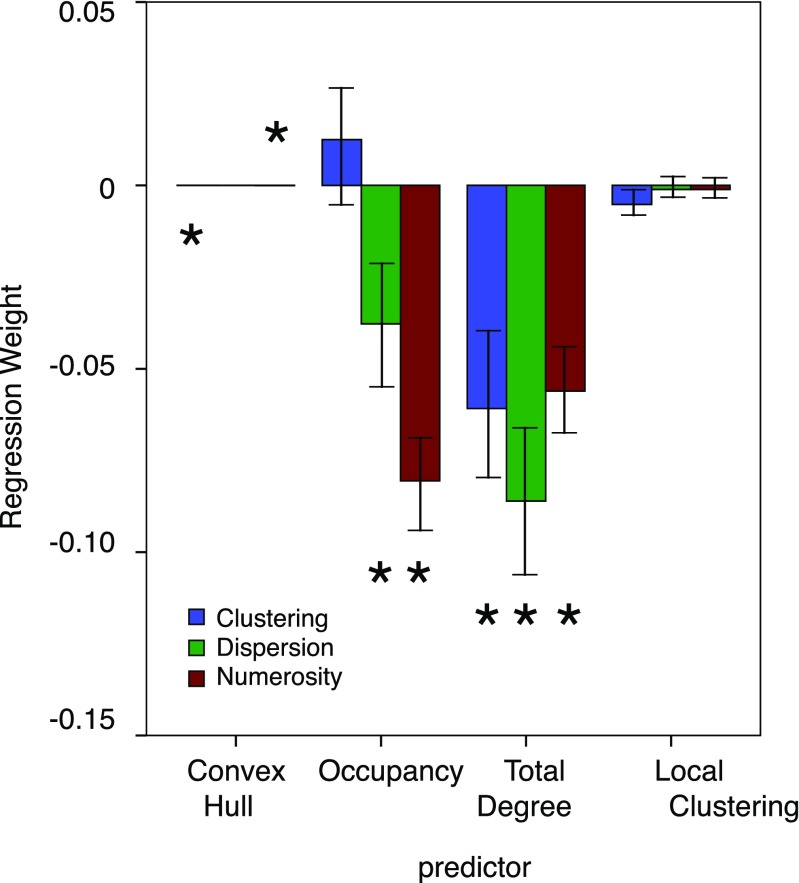
Regression weight as a function of task and predictor. The error bars depict SE of the mean. The asterisks indicate where a *t*-test (df = 23) demonstrated that the average value of the regression weight significantly differs from 0 (all *p* < 0.05). Please note that the regression weights for Convex Hull are several magnitudes smaller than for the other three predictors

A summary of the logistic regression fits can be found in Table 4. The regression weights for each of the four predictors are shown in Fig. 6.

**Table 4:** Overview of the logistic regression fits

Task	Percentile	χ^2^(4)	*p*	Nagelkerke R^2^
Numerosity	0 % (minimum)	0.75	0.000	0.00
	25 %	7.44	0.000	0.05
	50 % (median)	13.82	0.011	0.09
	75 %	23.17	0.118	0.15
	100 % (maximum)	51.23	0.945	0.30
Dispersion	0 % (minimum)	2.54	0.000	0.02
	25 %	13.41	0.000	0.09
	50 % (median)	28.12	0.000	0.17
	75 %	33.89	0.009	0.21
	100 % (maximum)	65.83	0.638	0.37
Clustering	0 % (minimum)	3.49	0.000	0.02
	25 %	10.86	0.000	0.07
	50 % (median)	18.06	0.001	0.12
	75 %	39.23	0.029	0.24
	100 % (maximum)	64.54	0.480	0.37

Task: task performed by participant. Percentiles (minimum, 25 %, median, 75 % and maximum) are shown for the i) the improvement in fit by adding the four predictors to the logistic model as measured by χ^2^; ii) the *p* value for this improvement; iii) the variance explained by the logistic model with four predictors (Nagelkerke R^2^).

We entered the values of the predictors into a Greenhouse-Geisser corrected 3x4 repeated measures ANOVA with task (Numerosity, Dispersion, and Clustering) and Predictor (Convex Hull, Occupancy, Total Degree, and Clustering) as independent variables. This ANOVA yielded main effects for task *F*(2, 46) = 4.0, *p* < 0.03, η^2^ = 0.148, and predictor *F*(3, 69) = 16.3, *p* < 0.001, η^2^ = 0.415. More importantly, there was a significant interaction between task and predictor *F*(6, 138) = 6.3, *p* < 0.001, η^2^ = 0.215. Because of this interaction, we performed separate one-way repeated measures ANOVAs for each of the predictors with task as the independent variable.

For Convex Hull, there was a significant effect of task *F*(2, 46) = 6.7, *p* < 0.003, η^2^ = 0.215. Follow-up *t*-tests revealed that the regression weight for Convex Hull in the Clustering task (3.8*10^−5^) was different from its regression weight in the Numerosity task (−2.3*10^−5^), *t*(23) = 3.5, *p* < 0.002. For Occupancy, there also was a significant effect of task *F*(2, 46) = 10.3, *p* < 0.001, η^2^ = 0.309. Follow-up *t*-test demonstrated a clear difference between the regression weights in the Clustering task (0.012) and the Numerosity task (−0.08), tI(23) = 4.3, *p* < 0.001. For Total Degree, there was no effect of task *F*(2, 46) = 1.2, *p* < 0.31, η^2^ = 0.050. This also held true for Clustering *F*(2, 46) = 0.6, *p* < 0.54, η^2^ = 0.027.

These additional analyses support the conclusion that, although the responses to the different tasks have something in common (Total Degree predicts performance in all, Local Clustering does not predict performance in any), they are differentially related to configural aspects of the patterns. Occupancy does not play a role in predicting clustering responses, and Convex Hull has no role in predicting the dispersion responses. Moreover, the regression weight for Convex Hull is positive in the clustering task but negative in the numerosity task. This clearly suggests that participants take the task into account when they make their choice, despite the fact that the patterns were identical for all three tasks.

## General Discussion

The visual system has a set of characteristics that define the appearance of stimuli based on factors, such as contrast, spatial frequency, and eccentricity. A simple way to think about complex tasks is that humans rely on some characteristic of the stimuli that correlates with the dimension to be estimated, especially when such estimates are computationally demanding. Put bluntly, if all you have is a hammer, then everything looks like a nail. Therefore, it may be that when asked about numerosity, dispersion, clustering, or other global properties of a large set of elements, human responses will always be highly correlated. At the opposite extreme of the possibilities is the idea that there are highly specialized mechanisms for specific properties, such as numerosity. Research on perception of numerosity has been extensive over the years, and its characteristics have been documented. For instance, we know that numerosity tends to obey Weber’s law (Dehaene & Changeux, 1993; Ross, 2003) and that there is a topographical representation of numerosity in the human parietal cortex (Harvey, Klein, Petridou, & Dumoulin, 2013). More relevant for our paper, we know that regularity of the configuration and spacing of the elements affects perceived numerosity (Ginsburg, 1980; Valsecchi, Toscani, & Gegenfurtner, 2013).

This study is the first attempt to compare different ways to measure structure within configurations of simple elements and relate these measures to perception of numerosity, perception of dispersion, and perception of clustering. Elements were placed at random within a circular region, without overlap. Convex hull is a measure of the size of the overall configuration, and it did help to predict (but only moderately) the judgements about the numerosity of the configurations. The occupancy area is the union of the areas occupied by a set of circular regions surrounding the elements (Allik & Tuulmets, 1991). This model therefore depends on a parameter, which is the radius of this region of influence. We compared nine radii, from twice to ten times the size of the element.

We expected occupancy to be more related to perceived numerosity than perceived dispersion or clustering. This was indeed the case. In addition, we found that the best predictor of perceived numerosity was a measure of occupancy computed for an intermediate value of the radius of influence. Our results may not allow us to pinpoint the precise value, but in the main regression this value was 5 times the radius of the elements, and it is interesting to note that this is the point where there was a maximum rate of change in the index (Fig. 5). The non-monotonicity may indicate a trade-off: increasing the radius captures more of the area of influence, but soon the cost of overlapping with other areas of influence is greater than the gain.

Total degree of connectivity and local clustering are both measures of clustering. The two are moderately correlated (0.44 in our sample of stimuli). Local clustering is more strongly correlated with the occupancy index, because it is more sensitive to the presence of multiple local clustering. In terms of human performance, total degree behaved more similarly to occupancy (they swapped roles as the main predictors for judgments of numerosity and of dispersion). Local clustering did not predict well either perceived numerosity or dispersion but was a useful predictor of perceived clustering (at least in the statistical regressions). This pattern supports the view that observers rely on different aspects of the configuration when estimating different but related dimensions: numerosity, dispersion, and clustering. Note that the comparison between tasks was based on data collected from the same set of observers and the same set of configurations (presented three times to each observer in different random order).

With respect to the procedure employed in our study, the forced-choice task proved very effective for our purposes. Two randomly generated configurations were shown in two intervals, with the same number of elements, and the task was to select the one that appeared more numerous. This design produces data that measure directly any bias in perceived numerosity related to aspects of the configuration and is promising for future studies on this and related research questions.

Our results support the proposal (Anobile et al., 2015) that the estimates of numerosity and of dispersion are based on different spatial information. However, our stimuli had relatively high density in terms of dots/deg^2^, and they were in the range for which Anobile et al. (2015) believe that texture-density mechanisms are at play. The numerosity was never higher than 40, but density varied between 0.67 and 1.23 dots/deg^2^. Because some aspects of our study were exploratory in nature, more research is necessary to explore the parameter space more systematically, including stimuli with lower densities.
